# Prenatal and acute cocaine exposure affects neural responses and habituation to visual stimuli

**DOI:** 10.3389/fncir.2015.00041

**Published:** 2015-08-25

**Authors:** Elizabeth Riley, Konstantin Kopotiyenko, Irina Zhdanova

**Affiliations:** ^1^Boston University Graduate Program in Neuroscience, Boston University School of MedicineBoston, MA, USA; ^2^Department of Anatomy and Neurobiology, Boston University School of MedicineBoston, MA, USA

**Keywords:** zebrafish, cocaine, prenatal drug exposure, visual processing, optic tectum, telencephalon, GCaMP-HS, calcium imaging

## Abstract

Psychostimulants have many effects on visual function, from adverse following acute and prenatal exposure to therapeutic on attention deficit. To determine the impact of prenatal and acute cocaine exposure on visual processing, we studied neuronal responses to visual stimuli in two brain regions of a transgenic larval zebrafish expressing the calcium indicator GCaMP-HS. We found that both red light (LF) and dark (DF) flashes elicited similar responses in the optic tectum neuropil (TOn), while the dorsal telencephalon (dTe) responded only to LF. Acute cocaine (0.5 μM) reduced neuronal responses to LF in both brain regions but did not affect responses to DF. Repeated stimulus presentation (RSP) led to habituation of dTe neurons to LF. Acute cocaine prevented habituation. TOn habituated to DF, but not LF, and DF habituation was not modified by cocaine. Remarkably, prenatal cocaine exposure (PCE) prevented the effects of acute cocaine on LF response amplitude and habituation later in development in both brain regions, but did not affect DF responses. We discovered that, in spite of similar neural responses to LF and DF in the TO (superior colliculus in mammals), responses to LF are more complex, involving dTe (homologous to the cerebral cortex), and are more vulnerable to cocaine. Our results demonstrate that acute cocaine exposure affects visual processing differentially by brain region, and that PCE modifies zebrafish visual processing in multiple structures in a stimulus-dependent manner. These findings are in accordance with the major role that the optic tectum and cerebral cortex play in sustaining visual attention, and support the hypothesis that modification of these areas by PCE may be responsible for visual deficits noted in humans. This model offers new methodological approaches for studying the adverse and therapeutic effects of psychostimulants on attention, and for the development of new pharmacological interventions.

## Introduction

Cocaine, a psychostimulant and widely used drug of abuse, interferes with visual functions during intoxication and withdrawal, with up to 50% of chronic cocaine abusers experiencing simple non-formed visual hallucinations (Siegel, 1978; Mitchell and Vierkant, 1991; Vorspan et al., 2012). The consequences of prenatal cocaine exposure (PCE) also include a variety of deficits in visual functions, including nystagmus (Spiteri Cornish et al., 2013), strabismus (Block et al., 1997) and reduced visual attention (Struthers and Hansen, 1992; Hansen et al., 1993; Heffelfinfger et al., 1997, 2002; Accornero et al., 2007; Ackerman et al., 2010), leading to increased risk of attention deficit and hyperactivity disorder (ADHD; Leech et al., 1999; Bandstra et al., 2001; Bada et al., 2011).

Visual perception is critical for processing conditional and unconditional stimuli, which are known to play a major role in drug addiction (Overton and Devonshire, 2008; Weiss, 2010).

Thus, cocaine’s effects on visual processing might also play an important role in the development of cocaine addiction and relapse, and could in part underlie the increased risk of drug abuse in humans exposed to cocaine prenatally (Delaney-Black et al., 2011; Richardson et al., 2013).

In studying PCE, a number of serious obstacles, including polysubstance abuse by most cocaine-using pregnant women, make it difficult to determine the specific effects of cocaine on human development (Ackerman et al., 2010). Animal models therefore present a unique opportunity to document the effects of PCE under controlled conditions and to study the mechanisms involved. We employ a zebrafish model, which has recently become a popular organism for studying the effects of drugs on brain functions and behavior (Gerlai et al., 2000; Guo, 2004; López-Patiño et al., 2008a; Thomas et al., 2009). Adult zebrafish respond behaviorally to acute cocaine administration (Darland and Dowling, 2001), displaying anxiety-like behavior and showing changes in brain dopamine and dopamine transporter levels following cocaine withdrawal (López-Patiño et al., 2008a,b). Moreover, our earlier study in developing zebrafish demonstrated that PCE interferes with gene expression and brain morphology (Shang and Zhdanova, 2007), including in the optic tectum, known to be the principal area of visual processing in zebrafish (Nevin et al., 2010).

In this study, we addressed whether, during early vertebrate development, acute or PCE, or a combination of the two, can interfere with processing of contrasting visual stimuli- wholefield red light flash (LF) and dark flash (DF). We assessed calcium concentration changes in neurons and their projections, a well-established indicator of neuronal activity (Muto et al., 2011; Rose et al., 2014) and using transgenic larvae expressing the calcium reporter GCaMP-HS, focused on two brain structures, the optic tectum and dorsal telencephalon (dTe).

The optic tectum, the zebrafish homolog of the mammalian superior colliculus, is a highly conserved visual structure, responsible for eye and head movement control and sensory integration, and contributing to visual attention. A series of studies support a view that hyper-responsiveness of the tectum is associated with increased distractibility and thus attention deficits, and that inhibition of tectal responses could underlie some of the therapeutic effects of psychostimulants in patients with ADHD (Clements et al., 2014). Recent studies also suggest that the optic tectum is involved in the pathways integrating visual information and its corresponding affective value, thus influencing affective biases in patients with mental disorders and addiction (Vuilleumier, 2014). Another principal component of these integration pathways is the cerebral cortex, critical for visual information processing and affected by psychostimulants, including cocaine (Muñoz-Cuevas et al., 2013). In zebrafish, a homolog of the mammalian cortex is the dTe (Mueller et al., 2011).

Here, we show that the optic tectum and dTe of a developing zebrafish have distinctly different patterns of response and habituation to contrasting visual stimuli. Cocaine can acutely modulate the responses to light but not darkness in this diurnal vertebrate. Importantly, we found that while prenatal exposure to cocaine alone does not modify neuronal responses to either visual stimulus tested, it induces tolerance to the effects of acute cocaine on visual signal processing later in life. Understanding the mechanisms through which acute or prenatal cocaine acts to interfere with visual functions, and which brain structures are affected, could help developing new treatment strategies for attention deficit and other consequences of early cocaine exposure.

## Materials and Methods

### Subjects

Wild-type (AB strain) and transgenic s1013t-GCAMP-HS larval zebrafish (Danio rerio) expressing the calcium indicator GCaMP-HS were used in this study. We bred the transgenic line from UAS:GCaMP-HS and s1013t lines. UAS:GCaMP-HS (Muto et al., 2011) was a generous gift from the laboratory of Koichi Kawakami, National Institute of Genetics, Japan. The s1013t line, a Gal4-VP16 line driving expression mainly in the optic tectum and telencephalon, was generated by the laboratory of Herwig Baier (Scott and Baier, 2009) and obtained from the Zebrafish International Resource Center (ZIRC, Oregon, USA). The two lines were crossed and F1 offspring expressing GCaMP-HS, but not kaede (present in s1013t line) were selected, raised and bred. Since F1 animals are heterozygous for GCaMP-HS, F2 larvae positive for GCaMP-HS were used for all neuroimaging experiments. Sex determination is not possible during the larval stage, thus sex is not indicated.

Adult male and female zebrafish (6 fish/3-L tank) were housed in a 14 h light/10 h dark cycle, in a temperature (27°C) and pH (7.0–7.4) controlled multi-tank re-circulating water system (Aquaneering, San Diego, CA, USA). Adult animals were fed three times a day with live brine shrimp (Brine Shrimp Direct, Ogden, UT, USA), enriched with fish pellets (Lansy NRD, Salt Lake City, UT, USA). Embryos and larvae were raised at 28°C in a 12 h light/12 h dark cycle, and fed paramecia three times a day starting at 5 days post fertilization (dpf). The experiments were conducted in 6–7 days old larvae, between ZT2 and ZT8 (ZT0 is lights-on time). All animal procedures were performed in accordance with the protocol approved by the Institutional Animal Care and Use Committee (IACUC) of Boston University School of Medicine.

### Prenatal Cocaine Treatment

For PCE, embryos were dechorionated with pronase (Sigma-Aldrich, St. Louis, MO, USA) at 24 h post fertilization (hpf) and then repeatedly exposed to a solution of 0.5 μM cocaine in egg water (60 μg/mL Instant Ocean salts in deionized water) at 24, 48 and 72 hpf. Each exposure lasted 15 min and was followed by a thorough washout of the drug, using a fine net to move larvae through five successive wells of fresh egg water, for 30 s each time. Controls were also dechorionated and treated with egg water. For details on acute cocaine treatment procedures, see “Experimental Design of Neuronal Imaging Studies” Section, below.

### Neuronal Population Imaging

At 7 dpf, s1013-t-GCaMP-HS larvae were immersed in 1.1 mM tubocurarine hydrochloride (Sigma-Aldrich, St. Louis, MO, USA) for 8 min to induce paralysis and prevent movement during imaging. Larvae were then embedded in approximately 12 μL of 1% low-melting agar (40°C, IBI Scientific, Peosta, IA, USA) and positioned dorsal side up in a glass petri dish. When the agar had set, the preparation was covered with egg water. Observation of a normal heartbeat and circulation in an embedded larva confirmed that it had not been injured by the procedure. A dish containing an individual larva was placed under the microscope and depending on protocol, adapted to darkness or 630 nm red light whole-dish illumination for 3 min. Two-photon imaging of both optic tectum hemispheres and the telencephalon was conducted simultaneously using a Zeiss LSM 710 NLO microscope with a Chameleon Vision S short-pulse pre-chirped titanium sapphire laser for multiphoton excitation at 930 nm using a 20 × NA 1.0 immersion objective. Zen software was used to operate the microscope. Care was taken to image at the same depth in the optic tectum and dTe each time, based on anatomical markers.

### Dark Flash and Red Light Flash in Neuronal Imaging Studies

The LF was an automatically generated 1-s 450-lux pulse of light produced by the 633 nM red laser triggered by the 2-photon microscope used for imaging. Between LF presentations larvae remained in total darkness. The DF was an automatic 1-s interruption in 450-lux 630 nM red light produced by 4 LEDs placed in a ring surrounding the embedded larva. A similar setup was used in both calcium imaging studies and behavioral studies described here.

### Experimental Design of Neuronal Imaging Studies

**Figure 1:**
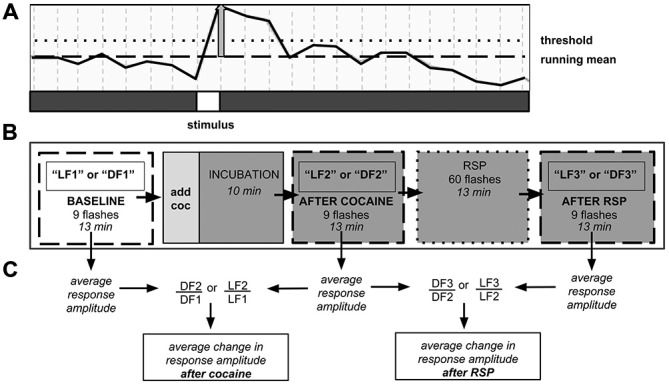
**Neuronal population imaging protocol. (A)** Detail of fluorescence signal from calcium reporter documented using two-photon confocal microscopy. Signal rises after presentation of the stimulus. Y-axis: fluorescence intensity. Vertical gray bar—amplitude of individual stimulus response. Responses that did not reach the threshold line were not counted. **(B)** Schematic of 3-trial imaging protocol. Each trial consisted of nine stimulus presentations over 13 min. **(C)** Schematic of data analysis. Change in signal amplitude was computed between trials. DF—dark flash, LF—light flash, LF1, DF1, etc.—light flash trial 1, dark flash trial 1, etc., RSP—repeated stimulus presentation.

In our within-subject design, each experiment consisted of three consecutive trials per fish, with each trial including 1000 confocal images taken every 750 ms over ~13 min (Figure 1). During each trial, 9 stimuli were presented. Following a 3-min adaptation to darkness or 630 nm red light, the first trial (LF1 or DF1) documented neural responses at baseline. Dark-adapted animals received red LF and light adapted animals received DF. Thereafter, the larva was exposed to either water (control) or cocaine (water in dish was carefully aspirated and replaced with 0.5 μM cocaine solution), followed by a 10-min incubation, to allow the drug to permeate the agar. Agar is porous (Schantz and Lauffer, 1962), and both previous experiments in our laboratory (Mabray, Unpublished doctoral dissertation), and the experiments described here, have shown that agar-embedded larvae display effects of cocaine within 10 min. Larval responses to DF or LF following cocaine administration were documented in the second trial (LF2 or DF2). Then the larvae were exposed to a repeated stimulus presentation (RSP), with 60 LF or DF stimuli over a 13-min period, with an inter-stimulus interval of 15 s. Time-control larvae remained in constant conditions. The third trial (LF3 or DF3) then allowed us to determine whether larvae had habituated or sensitized to RSP, and whether the presence of cocaine and/or prior PCE affected the results.

### Data Analysis for Neuronal Imaging Studies

Using custom software, regions of interest (ROIs) covering the optic tectum neuropil (TOn) and dTe were manually defined for each fish, based on anatomical boundaries. The average brightness of each region was then tracked throughout each trial (1000 images) and normalized to a 100-frame running mean centered around each stimulus presentation. Individual responses were defined as being above threshold if the increase in fluorescence was at least 1.5 standard deviations greater than the running mean, for at least one frame (0.75 s), immediately following a stimulus presentation. Response amplitude was reported as percent increase above running mean, and average RA for each stimulus train was computed. A 3% rise in signal amplitude was equivalent to a five-fold increase above background noise. Trials with movement artifacts due to incomplete restraint were not included in the analysis.

For each treatment group, the percent change in response amplitude between consecutive trials was reported (i.e., percent change between LF1 and LF2, and between LF2 and LF3). These percent change statistics were compared between treatment groups using linear mixed model analysis in SPSS (IBM, Armonk, NY, USA). Within groups, a one-sample *t*-test was used to determine whether response amplitude increased or decreased significantly between trials. Two-tailed *t*-tests were used to determine significance in the dose-response study. In all cases significance level was set at *p* < 0.05.

### Behavioral Assessment and Data Analysis

The locomotor responses to visual stimuli in zebrafish larvae, 6–7 dpf, were assessed using high-speed video recordings at 1000 frames per second (NAC Image Technology, Simi Valley, CA, USA), at 512 × 512 pixel resolution. All recordings were performed in a dark room, at 27 ± 1°C. Activity was recorded in groups of 20 larvae, in 51 mm petri dishes containing 15 mL of water. An infrared light (IR045, Wisecomm, Cerritos, CA, USA) was positioned 14 cm below the platform. The stage was illuminated by a custom built circuit of four red (660 nm) LEDs (276–015, Radioshack, Fort Worth, TX, USA) positioned at 15 cm above the dish. The light intensity at dish level was 290 lux (red light) or 0 lux (dark). LED circuit operation and the onset of camera recording were synchronized using a programmable electric pulse generator (Master-8, A.M.P. Instruments LTD; Jerusalem, Israel).

Depending on the experiment, each group was adapted to either red light or darkness for 8 min prior to assessment. Each test included 13 trials, each 800 ms in duration, with 60 sinter-trial interval. First, spontaneous activity in constant conditions (red light or dark) was evaluated (3 trials), followed by red light or dark flashes (LF or DF), accordingly (10 trials). Every 60 s, the constant condition (dark or red light) was interrupted by a 1000-ms LF or DF. The first 800 ms window of each 1000-ms LF or DF was captured for further analysis.

All kinematic analyses were performed using Flote 2.0 high throughput automated analysis software (Burgess and Granato, 2007b). Individual movements were scored using the Fourier analysis method with the angle threshold value of 2. Tracked larvae with anomalies in curvature and movement were excluded from analysis. Larvae that were moving at the very beginning of the trial, and larvae situated on the dish periphery, were automatically excluded from the analysis by Flote. The kinematic analysis results are presented as means of three consecutive trials for corresponding constant lighting conditions, or 10 consecutive trials for LF or DF presentation. Comparisons were performed using Student’s *t*-test (two-tailed, with unequal variance).

## Results

### Optic Tectum Neuropil Responds Differentially to Light and Dark Flashes, While Dorsal Telencephalon Responds Only to Light Flash

**Figure 2:**
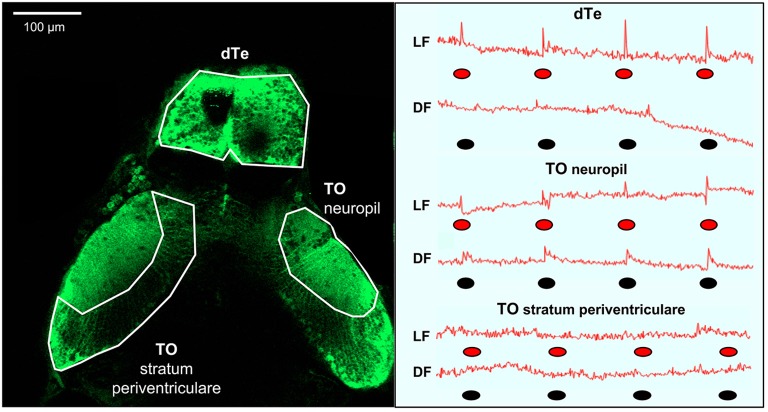
**Summary of light flash and dark flash responses in the optic tectum and dorsal telencephalon (dTe).** Left panel: Dorsal view of s1013t-GCaMP-HS larval brain (7 dpf) during two-photon confocal imaging. Anatomical boarders of the optic tectum (neuropil and stratum periventriculare) and the dTe are outlined. Right panel: Red line—traces of absolute fluorescence intensity during first trial and following exposure to red light (LF) or dark (DF) flashes, indicated by red and black ovals, respectively, for the optic tectum (neuropil and stratum periventriculare) and the dTe.

To investigate neuronal correlates of the LF- and DF-induced behaviors (Burgess and Granato, 2007a; Burgess et al., 2010), we used calcium imaging in transgenic fish carrying the genetically encoded calcium indicator GCaMP-HS, with fluorescence intensity providing a correlate of neuronal activity (Muto et al., 2011; Rose et al., 2014). Calcium responses were simultaneously monitored in two brain areas using two-photon confocal microscopy (Figure 2). In constant red light or dark, background fluorescence intensity in TOn and dTe generally did not deviate by more than 1%, consistent with prior characterization of the GCaMP-HS fluorophore (Muto et al., 2011). When the larva was exposed to LF or DF stimuli, a pronounced peak in fluorescence intensity power at the stimulus presentation frequency (0.012 Hz) was documented. All individual responses peaked within the first image frame following the stimulus (Figure 1). Response amplitude was reported as percent change in fluorescence intensity between running mean and response peak. In TOn, an increase in response amplitude was documented following each stimulus, whether LF (3.71 ± 0.38%) or DF (2.35 ± 0.19%). However, the duration of the response to DF (3.65 ± 0.34 s) was significantly longer than to LF (2.6 ± 0.23 s, *t*-test, df = 18, *p* = 0.026, Figure 2).

The dTe responded to LF with 4.78 ± 0.55% increase in response amplitude and to a 1.62 ± 0.08 s increase in duration, but no responses to DF could be documented in this region (Figure 2). No significant responses were observed to either DF or LF in the control region, TO stratum periventriculare.

### TOn Habituates to Repeated Dark Flash but not to Light Flash

To determine whether RSP can lead to habituation or sensitization of neural responses in either brain area studied, and whether these effects could be stimulus-dependent, we first conducted three 9-response “time control” trials (e.g., LF1, LF2, LF3), with a 13-min inter-trial interval (Figure 1). We then compared the results obtained to the results following RSP, a train of 60 stimuli with an inter-stimulus interval of 15 s administered between LF2/LF3 or DF2/DF3.

**Figure 3:**
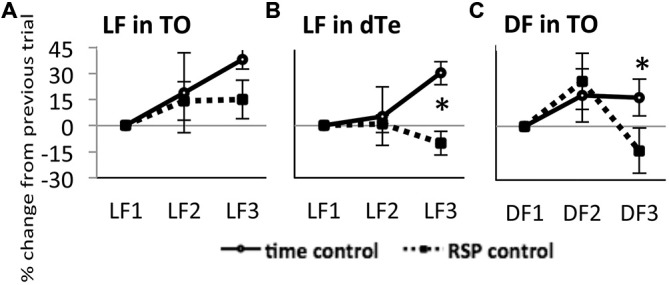
**Response of TOn and dorsal Te neuronal activity to repeated stimulus sresentation. (A)** Light Flash in TOn showing amplitude increasing over time Time-control (no RSP), *n* = 4, RSP-control, *n* = 17. **(B)** Light Flash in dorsal Te, showing habituation in RSP control. Time-control, *n* = 5, RSP-control, *n* = 15. *: linear mixed model, *n* = 53, *p* = 0.004. **(C)** Dark Flash in TOn, showing habituation in RSP control. Time-control, *n* = 5, RSP-control, *n* = 6. *: linear mixed model, *n* = 33, *p* = 0.028. LF1, LF2, LF3 separate trials. RSP exposure between LF2 and LF3 trials. All bars—group mean ± SEM.

In time control fish exposed to LF, the response amplitude in TOn showed a tendency to increase between LF1 and LF2 (22 ± 16%, NS) and exhibited a significant increase between LF2 and LF3 (37 ± 11%, *n* = 4, *p* = 0.002, Figure 3A). In dTe, LF response amplitude tended to increase between LF1 and LF2 (1 ± 5%, NS) and reached significance between LF2 and LF3 (30 ± 7%, *n* = 5, *p* = 0.006, Figure 3B). A similar trend was observed in TO in response to DF, though neither change reached significance (25 ± 16% between DF1 and DF2, and 16 ± 10% between DF2 and DF3, Figure 3C).

On this time-control background, showing a tendency towards increasing response amplitude between subsequent trials, the introduction of RSP between the second and third trials led to further changes in response amplitude. In TOn, response amplitude to LF increased after RSP (15 ± 11%, NS, Figure 3A). In contrast, in dTe, response amplitude decreased after RSP (−10 ± 7%, *n* = 53, *p* = 0.004 vs. time-control, Figure 3B). TOn responses to DF also diminished between the second and third trials (−14 ± 13%, *n* = 33, *p* = 0.028 vs. time-control, Figure 3C). There was no dTe response to DF over these trials. Together, these results suggested that TOn habituated to DF but not LF, while dTe habituated to LF. These groups, receiving RSP in the absence of any drug treatment, later served as “RSP control”.

### Acute Cocaine Exposure Reduces Light Flash Response Amplitude in TOn and dTe, but Prevents Habituation to Repeated Light Flash in dTe

**Figure 4:**
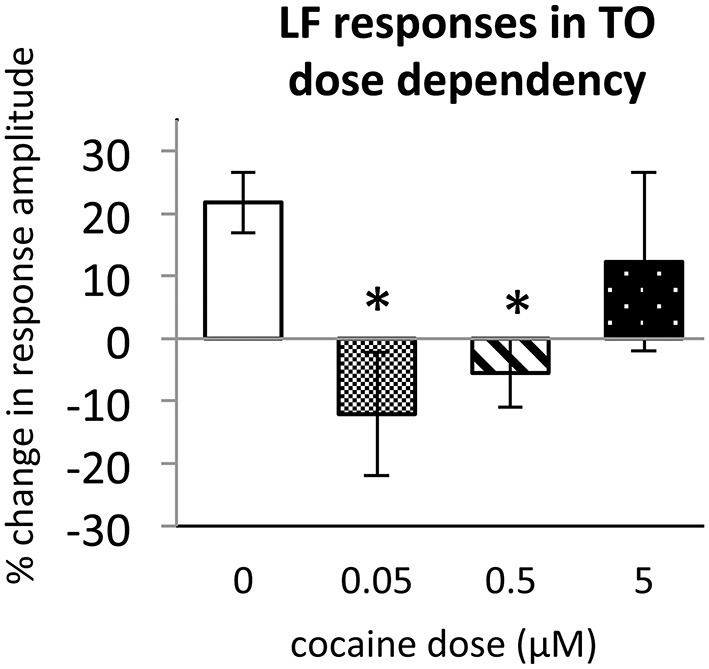
**Dose dependence of cocaine effects on Light Flash response in TOn.** Cocaine concentrations: 0 μM, *n* = 17; 0.05 μM, *n* = 8; 0.5 μM, *n* = 16; 5 μM, *n* = 10. All bars—group mean ± SEM. *T*-test between 0 μM and 0.05 μM, df = 29, *p* = 0.001. *T*-test between 0 μM and 0.5 μM, df = 23, *p* = 0.003. *p < 0.05.

**Figure 5:**
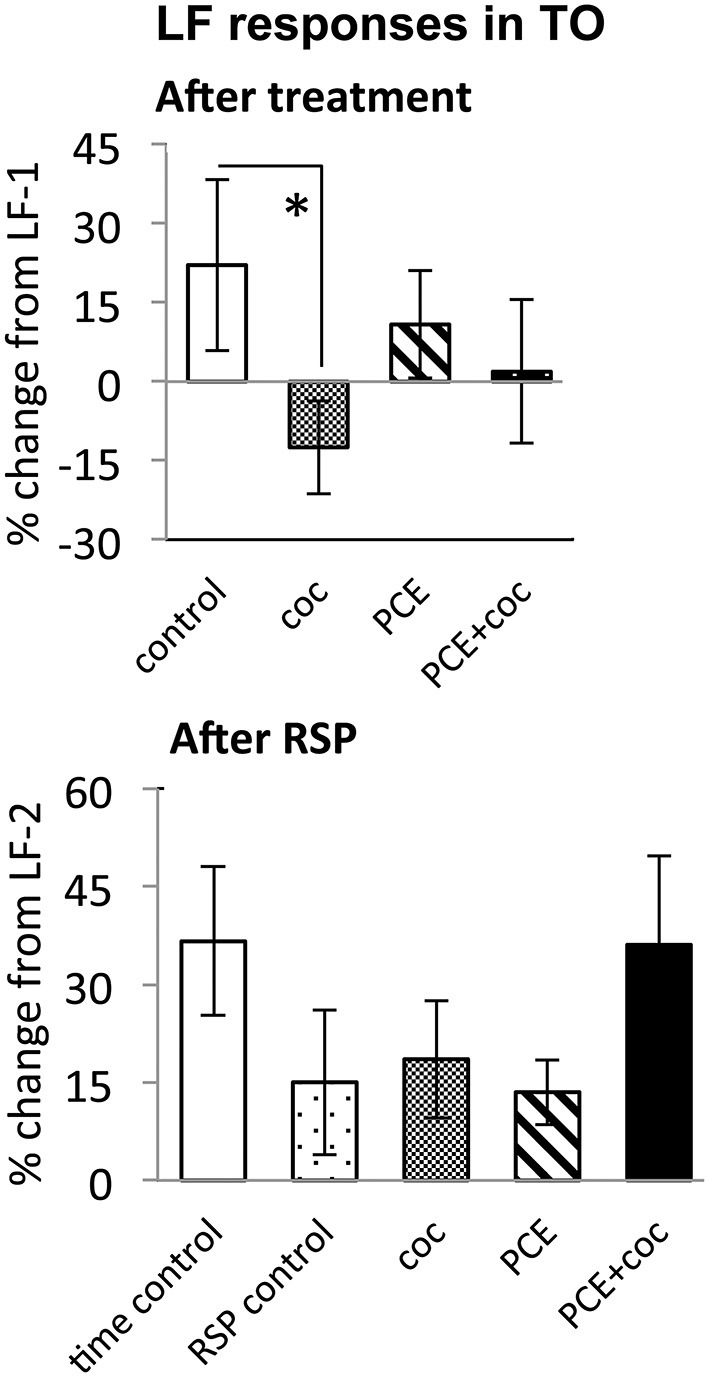
**Effects of cocaine, PCE, and PCE + cocaine exposures on LF responses in TOn, and modification of effects of RSP by cocaine.** **(A)** Decreased response amplitude after cocaine in drug-naive fish. *: linear mixed model, *n* = 25, *p* = 0.049. **(B)** No signficant effects of cocaine. RSP—repeated stimulus presentation. All bars—group mean ± SEM. Time-control, *n* = 4; RSP-control, *n* = 17; coc, *n* = 12; PCE, *n* = 12; PCE+coc, *n* = 10.

**Figure 6:**
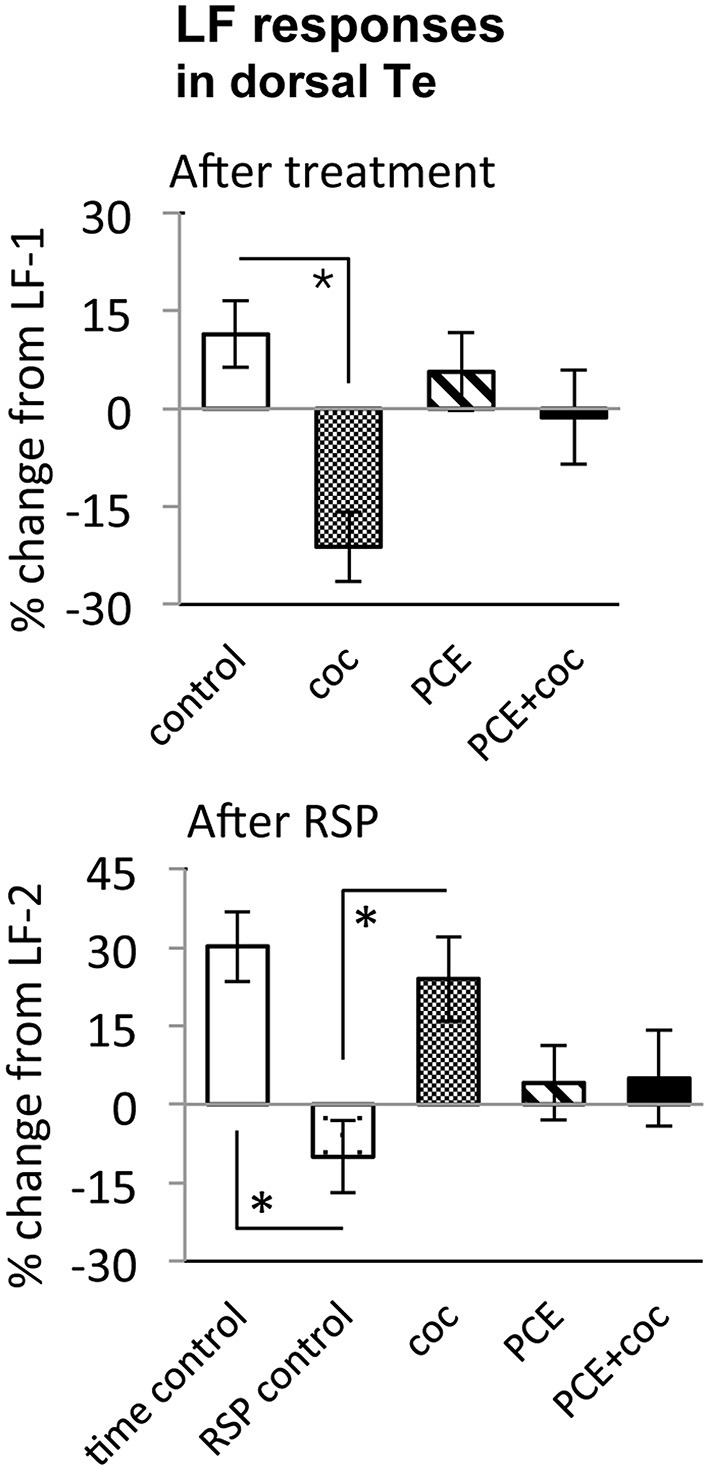
**Effects of cocaine, PCE, and PCE + cocaine exposures on light flash responses in dorsal Te, and modification of the effects of RSP by cocaine.** **(A)** Decreased response amplitude after cocaine in drug-naïve fish. *: linear mixed model, *n* = 53, *p* = 0.018. **(B)** Habituation after RSP in control fish, but not in drug-naïve cocaine-treated fish. *time control vs. RSP control: linear mixed model, n = 53, *p* = 0.004. *RSP control vs. coc: linear mixed model, n = 53, *p* = 0.002. RSP—repeated stimulus presentation. All bars—group mean ± SEM. Time-control, *n* = 5; RSP-control, *n* = 15; acute only, *n* = 10; PCE-only, *n* = 12; PCE and acute cocaine, *n* = 11.

To determine the effect of acute cocaine exposure on neuronal responses, we administered cocaine to the recording chamber of individual larvae between the first and second trials (Figure 1B). The LF response was sensitive to acute cocaine. The dose dependence study showed that, in TOn, the response amplitude to LF decreased significantly between the first and second trial during exposure to either 0.5 or 0.05 μM cocaine, but not 5 μM cocaine (*n* = 31 and 25, *p* = 0.001 and 0.003 respectively, Figure 4). This is consistent with the previously observed bimodal dose effects of cocaine in larval zebrafish (Shang and Zhdanova, 2007). Based on this, the 0.5 μM cocaine dose, which is similar to those that a human fetus with PCE is typically exposed to Jones et al. (2008) and De Giovanni and Marchetti (2012), and led to the least inter-individual variability in our assays, was used for all further experiments. This concentration evoked a 12 ± 9% decrease in TOn response amplitude (*t*-test, df = 23, *p* = 0.049, vs. control, Figure 5) and a 21 ± 5% decrease in dTe response amplitude (*n* = 53, *p* = 0.018, vs. control, Figure 6).

In TOn, no habituation was observed following RSP in either control or cocaine conditions (Figure 5). In dTe, however, following RSP the response amplitude to LF increased by 24 ± 8% (*n* = 53, *p* = 0.002, vs. control), in contrast to the habituation that had been observed in control animals (Figure 6).

### Acute Cocaine does not Change Responses to Dark Flash in TOn

**Figure 7:**
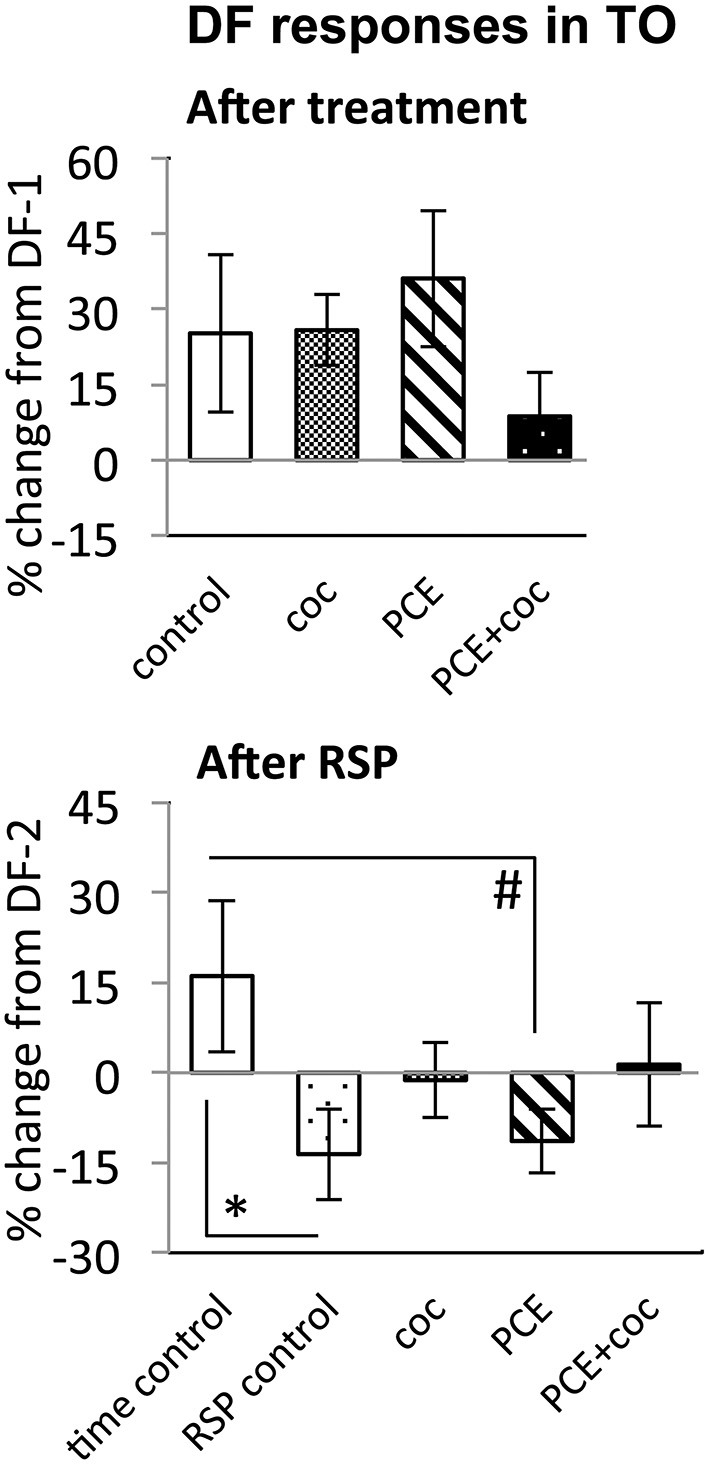
**Effects of cocaine, PCE, and PCE + cocaine exposures on DF responses in TO, and modification of the effects of RSP by cocaine.** **(A)** No significant effects of cocaine. **(B)** Habituation after RSP in control and PCE fish only. *: linear mixed model, *n* = 33, *p* = 0.028. #: linear mixed model, *n* = 33, *p* = 0.023. RSP—repeated stimulus presentation. All bars—group mean ± SEM. Time-control, *n* = 5; RSP-control, *n* = 6; acute only, *n* = 8; prenatal only, *n* = 8; acute and prenatal, *n* = 6.

The DF response in TOn was not sensitive to acute cocaine exposure. Similar to control fish, in which response amplitude to DF increased between the first and second trial, cocaine-treated fish showed a 25 ± 16% increase in response amplitude (NS vs. control, Figure 7).

While time-control and RSP-control groups differed significantly from each other (*n* = 33, *p* = 0.028, Figure 7), reflecting efficacy of RSP, animals treated acutely with cocaine did not differ significantly from either control group, suggesting a trend for cocaine to attenuate habituation to DF in TOn.

### Prenatal Cocaine Exposure Makes Larvae Insensitive to the Effects of Acute Cocaine

To determine whether PCE administered repeatedly at 24, 48 and 72 hpf would modify visual responses or the outcome of acute cocaine treatment in 7 dpf larvae, we documented the effects of PCE alone, and the effects of acute cocaine in PCE larvae. PCE alone did not significantly affect response to DF or LF in either of the brain structures studied (Figures 5–7). However, in contrast to drug naive larvae, following acute cocaine administration PCE larvae did not show a reduction in LF response amplitude in either TOn or dTe (Figures 5, 6). Moreover, acute cocaine treatment no longer prevented habituation to LF in dTe of PCE larvae, in contrast to drug naïve animals (Figure 7).

### Locomotor Activity in Darkness and Response to Dark Flash Differ from those in Red Light or in Response to Red Light Flash

**Figure 8:**
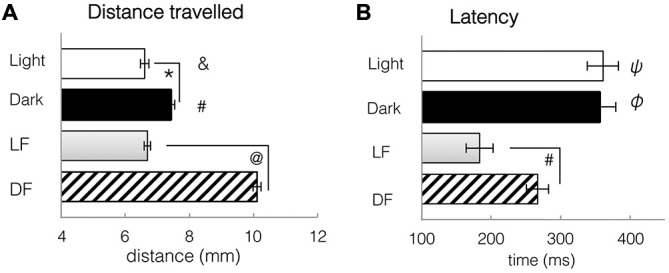
**Spontaneous locomotor activity under constant conditions of light and dark, and kinematic responses to light flash and dark flash. (A)** Distance travelled during a single trial, either spontaneously in constant conditions, or in response to LF or DF. Larvae swim further in dark or after DF than in light or after LF. *T*-tests: *: spontaneous light vs. spontaneous dark, df = 310, *p* = 0.0061. #: spontaneous dark vs. DF, df = 706, *p* = 2.3 × 10^−7^. &: spontaneous Light vs. DF, df = 700, *p* = 2.7 × 10^−8^ @: LF vs. DF, df = 1051, *p* = 7.34 × 10^−8^. **(B)** Latency to first movement, either spontaneously in constant conditions, or in response to LF or DF. Latency to movement is decreased after stimulus presentation, especially LF. *T*-tests: ψ: spontaneous light vs. LF, df = 655, *p* = 6.97 × 10^−5^. φ: spontaneous Dark vs. DF, df = 706, *p* = 0.0035 #: LF vs. DF df = 1051, *p* = 0.0011. All bars—group mean ± SEM. DF—dark flash, LF—light flash.

To image neuronal activity using infrared two-photon microscopy, we had to use red LF in order to prevent damage to the microscope’s sensitive detector. To provide valid comparison to earlier studies that used white LF vs. DF for studying larval behaviors, we documented locomotor activity over 13-min periods of constant darkness or red light, and then following DF or red LF. We found that the s1013t-GCaMP-HS transgenic larvae, as well as wild-type fish, were both spontaneously more active in constant darkness than in red light (*t*-test, df = 310, *p* = 0.0061, Figure 8A). This was similar to results reported in other strains exposed to white light vs. darkness (Burgess and Granato, 2007a).

Following either red LF or DF, latency to movement was significantly reduced when compared to spontaneous swimming in continuous red light or dark (*t*-tests, LF: df = 655 groups, *p* = 6.97 × 10^−5^; DF: df = 706, *p* = 4.8 × 10^−6^, Figure 8B). Locomotor responses to LF were significantly faster than to DF, with significant difference in latency to movement after LF (183 ± 20 ms) than after DF presentation (266 ± 6 ms, *t*-test, df = 1051, *p* = 0.0011, Figure 8B). Activity patterns were not significantly modified by LF, in comparison to swimming in constant light. However, DF resulted in pronounced changes in behavior, e.g., increased bend angle (*t*-test, df = 706, *p* = 4.8 × 10^−6^) and significantly greater swim distance when compared to spontaneous swimming in dark (*t*-test, df = 706, *p* = 2.3 × 10^−7^, Figure 8A) or light (*t*-test, df = 700, *p* = 2.7 × 10^−8^, Figure 8A). These results, similar to previously published work extensively characterizing larval zebrafish activity in white light and in darkness (Burgess and Granato, 2007a), suggest that larval locomotor responses to red light and white light are similar, and both differ from responses to darkness or dark flash.

## Discussion

Our principal findings demonstrate region-specific responses to contrasting visual stimuli in the developing brain, stimulus-specific alteration of these responses by acute cocaine, and the ability of PCE to modify visual responses to cocaine later in life. We found that the optic tectum responds to both light flash and dark flash, while dTe responds only to light flash. Acutely, cocaine inhibits responses to light flash in both the optic tectum and dTe, but only interferes with adaptation to RSP in the dTe. Importantly, PCE produces no measurable effects on its own but prevents the inhibitory effects of cocaine on visual perception later in life.

These results are important in light of the major role that the optic tectum, in connection with the cerebral cortex, plays in sustaining visual attention, and the significant changes in attention induced by PCE in humans. Our findings further support the notion that the optic tectum mediates these prenatal cocaine-induced changes in attention (Overton, 2008) and establishes a new approach to studying the neurochemical circuits underlying both the adverse and therapeutic effects of psychostimulants on attention.

### Effects of Cocaine on the Optic Tectum Neuropil are Stimulus- and Region-Dependent, and Relevant to Attention

Our choice of the optic tectum—the superior colliculus in mammals—as one of the brain structures of interest was based on its critical role in visual signal processing, control of eye and head movements and visual attention (Overton, 2008; Nevin et al., 2010; Krauzlis et al., 2013). It has been known for some time that superior collicular lesions attenuate distractibility in cats (Sprague and Meikle, 1965), rats (Goodale et al., 1978) and monkeys (Milner et al., 1978). Consistent with this, in zebrafish, pharmacological augmentation of the tectal response to light flash, using bicuculline, is associated with decreased ability to catch prey, which requires a high level of visual attention and coordination (Del Bene et al., 2010). Tectal light flash responses are thought to represent active filtration of whole-field visual information by the superficial layers of the tectum. The deeper layers of the tectum are then better able to track moving objects such as prey (Del Bene et al., 2010). Importantly, mammals also exhibit layer-specific filtration of visual information in the superior colliculus (Isa, 2002). These collicular effects are highly conserved and humans manifest increased distractibility after the loss of inhibitory control of the prefrontal cortex over the superior colliculus (Gaymard et al., 2003).

In our zebrafish model, cocaine suppresses responses to light flash in the superficial layer of the optic tectum. Similarly, another psychostimulant, amphetamine, suppresses responses to light flash in the superficial layer of the superior colliculus in mammals, and this is associated with increased visual attention (Gowan et al., 2008). These important observations led to a hypothesis that ADHD and perhaps other conditions of increased distractibility are associated with a hyperactive superior colliculus, and that consequently, reducing superior colliculus activity can attenuate distractibility, contributing to the therapeutic effects of psychostimulants in ADHD (Comoli et al., 2003; Overton, 2008; Clements et al., 2014).

In this context, we were especially interested to discover that PCE, in doses similar to those that human fetus can be exposed to *in utero* (Jones et al., 2008; De Giovanni and Marchetti, 2012), does not interfere with basal visual responses of the zebrafish optic tectum but prevents it from being modulated by acute cocaine later in life. This unexpected finding suggests that the optic tectum target of psychostimulants probably play a regulatory function, involved in modulating rather than generating visual responses. Identifying these supposed regulatory targets could improve the design or discovery of treatments for PCE or other types of increased distractibility resistant to psychostimulant treatment.

Indeed, the superior colliculus contains all three monoaminergic targets for psychostimulants. Of the three, the superficial layer of the superior colliculus, corresponding to TOn, is most densely innervated by noradrenergic and serotonergic afferents (Parent et al., 1981; Wichmann and Starke, 1988), with fewer dopaminergic projections (Weller et al., 1987; Campbell et al., 1991; Parker et al., 2013). However, the deep layers of the superior colliculus project directly to the dopaminergic neurons of the midbrain (Comoli et al., 2003), and can activate them in response to visual stimuli (Dommett et al., 2005). Thus, despite the relative lack of dopaminergic fibers in the superior colliculus itself, there is strong evidence that a hyper-responsive superior colliculus could contribute significantly to the dopaminergic dysregulation seen in increased distractibility.

Our findings also highlight the differences between the optic tectum responses to light and dark stimuli. While the optic tectum is activated by both light and dark flashes, repeated presentation of dark flash results in habituation, while no such effect is observed following repeated light flash. This disparity, and the fact that, in contrast to light flash, response to dark flash is not modulated by acute or PCE, suggest that distinct mechanisms are involved in processing light and dark stimuli in the optic tectum. This notion is strongly supported by an earlier study showing that dark flash stimuli are received and processed by the OFF retinal pathway, leading directly to turn movements which cause the larva to navigate away from dark regions (Burgess et al., 2010). In contrast, light flash stimuli, as shown by the same study, are processed by the ON retinal pathway, and result in activation of serotonergic neurons which then allow the larva to navigate towards the stimulus.

### Visual System Alterations Following Prenatal Cocaine Exposure and Tectal Dysfunction

Initial reports on cognitive deficits in preschoolers exposed to cocaine prenatally (Azuma and Chasnoff, 1993) have been further supported by work documenting cognitive and behavioral problems in their school-age and teenage counterparts (Ackerman et al., 2010; Buckingham-Howes et al., 2013). Extending from infancy through young adulthood, however, this population shows pronounced deficits in visual attention which may contribute significantly to their behavior problems (Struthers and Hansen, 1992; Hansen et al., 1993; Heffelfinfger et al., 1997, 2002; Accornero et al., 2007; Ackerman et al., 2010). Moreover, given the effects of psychostimulants on the superior colliculus (Gowan et al., 2008), it is not surprising that several specific visual system problems noted in children exposed to cocaine prenatally are consistent with collicular dysfunction, including nystagmus (Spiteri Cornish et al., 2013), strabismus (Block et al., 1997), and poor visual memory (Struthers and Hansen, 1992; Hansen et al., 1993).

Based on earlier findings linking a hyper-activated optic tectum and increased distractibility (Overton, 2008), which is also observed following PCE (Bandstra et al., 2001; Delaney-Black et al., 2011), we therefore expected that prenatal exposure to cocaine in zebrafish embryos would lead to altered activity of the superficial layers of the optic tectum. The results were different than expected. There were no significant changes in the baseline responses of the optic tectum to light flash or dark flash after PCE. However, when prenatally exposed larvae were challenged with a dose of acute cocaine later in development, they demonstrated a loss of the inhibitory effects of cocaine on tectal response to visual stimuli, suggesting that they had developed tolerance to the psychostimulant. This unexpected discrepancy may allow future studies to locate targets of PCE that do not affect the visual response itself but rather its modulation by psychostimulants. Studying this phenomenon further might also lead to an increased understanding of the mechanisms behind the therapeutic effects of psychostimulants on increased distractibility, or help to develop novel pharmacological treatments lacking potential for abuse.

### Selective Response to Light Flash in Dorsal Telencephalon and Its Modulation by Cocaine

The dTe in zebrafish is homologous to the cerebral cortex in mammals (Mueller et al., 2011; Ng et al., 2012) and to our knowledge, its visual response properties have not been investigated until now. We find that dTe is responsive to light flash only, since it did not respond to dark flash under any of the conditions tested here, and that cocaine acutely inhibits this response, as it does in the optic tectum. Importantly, cocaine prevents habituation of the dTe to repeated light flash. This is in striking contrast to the optic tectum, in which habituation to light flash does not develop, and acute cocaine has no impact upon adaptation to RSP. Both inhibition of the light flash response and inhibition of habituation to it by acute cocaine are prevented in the dTe by PCE.

Neither the specific functions of the zebrafish telencephalon, nor its homology to mammalian cortex have been explored well enough to precisely interpret the telencephalic response that we observed. However, clues regarding its function can be obtained from our behavioral observations. We find that larval zebrafish respond more rapidly to light flash but with more pronounced locomotor behaviors following a dark flash, including increased bend angle and distance traveled. This has been observed in earlier studies and explained by the need to navigate away from dark towards preferred lighted environments (Burgess and Granato, 2007b; Burgess et al., 2010; Steenbergen et al., 2011). The diurnal lifestyle of zebrafish larvae, which are active during the day and sleep at night (Zhdanova et al., 2001), necessarily means that bright light is associated with more complex visual processing related to a broad repertoire of daytime individual and social behaviors (Burgess and Granato, 2007a). Light is critical for prey capture, a behavior well developed in our 7 days post fertilization larvae, and one that requires exceptional attention and motor coordination (Borla et al., 2002; Gahtan et al., 2005; McElligott and O’malley, 2005). Thus, the fact that light flash but not dark flash activates dTe neurons might reflect the role of this structure in higher-level signal processing and/or learning and memory functions which are much less engaged when a diurnal animal is in dark environment.

Overall, as demonstrated here, the zebrafish model has the potential to significantly contribute to our understanding of the brain region-specific and stimulus-specific effects of acute and PCE. Early development of sophisticated visual responses and the critical role of attention in prey capture activities make the zebrafish an excellent model to uncover the mechanisms of adverse and therapeutic effects of psychostimulants on vertebrate brain and behavior.

## Conflict of Interest Statement

The authors declare that the research was conducted in the absence of any commercial or financial relationships that could be construed as a potential conflict of interest.
